# The Future of Medicine1Oration in Medicine, delivered at the annual meeting of the Illinois Medical Society, in Danville, May 18, 1910.

**Published:** 1910-11

**Authors:** Charles G. Stockton

**Affiliations:** Buffalo, N. Y.


### The Future of Medicine[1]

BY CHARLES G. STOCKTON, M.D.
Buffalo, N. Y.

(*Illinois Medical Journal*, July, 1910.)

THE topic of these remarks was suggested in a recent personal letter from a physician of Boston, as one suited to an occasion like this. The questions that are raised are familiar subjects of discussion between physicians. They receive the consideration of medical societies, editorial attention in journals, and they concern the sociologist as much as they do the physician. Perhaps the best way to introduce the matter is to present this recent instance, for although other examples crowd about us, this particular one was the source of the discussion that led to the writing of this paper.

1. Oration in Medicine, delivered at the annual meeting of the Illinois Medical Society, in Danville, May 18, 1910.

The Boston Medical Society has issued the following appeal, from which I abstract the greater portion.

*To the Superintendents and Trustees of the Various Hospitals of Boston:*

WHEREAS, The dispensary evil, or indiscriminate medical charity, in all the hospitals, clinics and dispensaries of Boston, is being intensified and aggravated from year to year.

WHEREAS, Large numbers of patients suffering from chronic diseases, acute illnesses and minor surgical injuries, undoubtedly able to pay a fee of some kind to a private physician or surgeon, are daily accepted and treated without adequate question or investigation as to their prosperity or pauperism, practically entirely free of charge.

WHEREAS, It has become a widespread habit of thought, wholly; or, largely because information or instruction to the contrary at the hospitals and clinics has never been systematically and officially given out, that any case of surgery or minor surgery can only be properly treated at some hospital or dispensary, thus depriving all ordinary private physicians and surgeons of a class of cases in the community which they have justly anticipated would come to their offices for treatment.

WHEREAS, The friendly and mutually advantageous relationships between the general practitioner and the consultant and specialist are often frustrated by the promiscuous, gratuitous services given by the latter at the ever-increasingly popular clinics.

WHEREAS, The "district physicians" sent out from the Boston Dispensary (who disclaim being city doctors), with their assistant nurses, have their headquarters at reliable druggists, and who give their services free, exemplify a form of medical practice which at times the ordinary practitioner has to compete with at a disadvantage.

WHEREAS, The Boston Lying-in Hospital, with its present monopolistic policy of accepting cases of obstetrics at rates and with facilities which defy all competition on the part of the private physician, excluding as it does nearly all impecunious cases and taking in practically only those well able to pay, constitute a hardship and an ethical wrong not easy of acceptance to the medical profession.

WHEREAS, The present unfair and inequitable system in vogue at the Massachusetts General Hospital, the Boston City Hospital and the relief stations, the Infants' Hospital on Blossom Street, the Boston Dispensary, the Lying-in Hospital, the Carney Hospital and many other clinics, of accepting daily large numbers of patients of all kinds and without seemingly sufficient discrimination, renders it extremely difficult if not impossible for the average practitioner to successfully obtain even a fair living practice, in competition with these influential institutions, the charters of which were granted to them in the interest of charity for the worthy poor.

*Resolved,* That the general policy of the hospitals, dispensaries and medical institutions, both public and semi-private, fails at the present time to recognise the principles and sources of professional existence, ordinary medical *esprit de corps,* or even the square deal, thereby inculcating in the minds of the laity a lack of confidence in the abilities of the ordinary private practitioner and a disrespect for his methods of practicing the art of medicine, and monopolising and injuring the legitimate opportunities in the community of the private practice of medicine and surgery.

*Resolved,* That these resolutions be sent to the several hospitals and dispensaries of Boston, with the request that hearings be given by the trustees, superintendents and hospital staffs on the general subject.

We all know that the statements made in this letter are true, or, if not so in Boston, the general facts are true in many other cities. The profession of medicine having found it necessary to educate the public as to the problems of disease, the general public has, to a considerable extent, associated itself with physicians in combating the same. The movement of the American Medical Association seeking to enlist the interest and assistance of the laity in disseminating through organised efforts a knowledge of public and personal hygiene, under the guidance of a special committee, may be cited as one of the forces at work in stimulating communities to unite with physicians in preventing and managing disease. Consciously and unconsciously, we have been arousing the public to the realisation of a great civic and humanitarian duty; we have shown foresight in pointing out the stupidity and the wastefulness of permitting recognised causes of preventable diseases to go unchecked. Daily encounter with helpless suffering stimulated us to demand the establishment of hospitals and varied institutions for the relief of the poor. We have justly insisted upon the necessity of these for the comparative study of disease and for providing the most economical means of relief. We have sought the advice and financial support of the community and have gained this through continued educational efforts. We are rewarded in some degree for our labor, and a more general response to our active propaganda is most probable. Never before has there been displayed such a widespread interests in matters of public health, and results are sprouting out in new and perhaps unexpected directions. We are seeing upon every hand the creation of tuberculosis camps and clinics; hospitals for incipient cases and for advanced cases. In the cities there is an increasing number of milk-distributing stations with free teaching and advice by salaried physicians for the prevention and treatment of sick children.

One of the most interesting and important developments in late years is a social service department connected with the great hospitals and other charities by means of which the unfortunate and ignorant are shown the path to healthful and happier lives. In many places there are school physicians whose service, if carefully performed, is certain to result in the decrease of contagious diseases and in correcting disability in children before disease has become thoroughly established in them. Tenement-house inspection and social settlement work are spreading, public playgrounds are provided and the necessity of pure air insisted upon. In a similar spirit school nurses are employed; in railway stations and various terminals nurses are coming to be a necessary addition to the force, and these nurses are in close touch with hospitals and relief stations which look out for unfortunate travelers. Great department stores, great factories employ their physicians and nurses, and we are now hearing of steps taken by insurance companies to instruct their assured as to the best manner to avoid illness and injury. In some instances nurses are supplied free by the companies for the benefit of the policy holders. We may foresee that in the near future expert physicians will be employed by companies in a similar function. An association insuring people of small incomes against sickness and disability has recently proposed to employ a group of expert physicians who should take charge of the policy holders during illness. The physicians are to be well paid by the company and the latter expects to be largely the gainer through the shortening of the period of illness and avoidance of malingering.

Within the recollection of some men present the insane were cared for in prisons or at home under the charge of the family physician and the practical nurse. Today sufferers from mental diseases are practically all cared for in institutions, and for the most part these are provided by the state or municipality. We find that the cities are now building well-equipped hospitals for contagious diseases, and the majority of patients in these institutions are cared for by the physician in charge of the hospital. This results not only in economy in nursing, isolation, and the like, and prevents the spread of disease, but materially lessens the occupation of the family physician. Besides, there are growing up psychopathic institutions, hospitals for the neurasthenic, which are practically free for those who are supposed to be indigent.

The state has provided gratuitously antitoxin serum for the treatment of diphtheria, serum for the treatment of rabies, and most likely it will soon provide sera for the treatment of various forms of meningitis and other preparations for the benefit of those who need potent and reliable means of combating terrific diseases. In the State of New York a bill has been introduced in the legislature asking for the gratuitous providing to midwives and physicians of preparations for the prevention of ophthalmia neonatorum. The object, of course, is to do away with a large number of blind, the majority of whom lose their vision through this preventable disease.

I must not catalogue the increasing number of movements springing up on every hand for the education of the people in the matter of hygiene for the prevention of disease, and offering assistance to those seeking restoration of health. Not one of this group fails in its humane purpose. There is scarcely one that physicians would not approve of, and yet the effect of all this upon the work and income of the average family physician is a matter worthy of the deepest consideration. To a different class from the institutions just cited belongs the work of the lodge doctor, the contract physician, those who care for the sick and injured in mines and factories, railroads, and the like, and at salaries pitifully small compared with the number of patients seen, the responsibility assumed and the arduous work done. The practice of such men we have regarded as distinctly antagonistic to the welfare of all concerned. In some form this is likely to continue, but surely the service should be improved and the physicians better paid. Somewhat analogous is the work of the city district physicians who are appointed through political influence and who are supposed to care for the poor without charge. Such physicians should be civil service appointees and should be adequately compensated. It seems to me proper to place this group in a class by itself in our consideration of this subject.

Let us see where this is leading us. For instance, consider the ludicrous position of the doctors of a certain great city, who for a decade have been fighting to obtain a pure water supply in order that typhoid fever might be eradicated from their midst. At last the pure water supply has become a reality and typhoid fever has disappeared, with the result that a numerous group of physicians who depended largely upon typhoid for their income are ruined.

This is not a problem confined to America, for, as it is the legitimate offspring of science and enlightenment, it follows that the problem confronts the profession of Europe as well as America. In England, where modern ideas of sanitation have proceeded farther, the pinch is more keenly felt than here, and the journals bear testimony to the evident embarrassment felt by the general practitioner in his effort to support his family and maintain his position.

Another line of thought must be considered here. Today a man finds himself incompetent to practise his profession without long training, a probational period in hospital life and opportunities placing him in touch with well-equipped laboratories and clinics in order that he may be in accord with the changing conception of pathology and novel methods of technic. The days of the "saddle bags" and the "medicine shelf" have disappeared, in place of which the physician must have his microscope, his incubator and his chemical reagents. He must provide himself with an easy access to the *x*-ray apparatus, and he must combine in himself the art of the expert mechanic, the physicist, the chemist, and the technician without losing one iota of his clinical sense and judgment. In short, the requirements of the modern physician are increasing in an inverse ratio with the number of patients and the sources of income; and this at a time when the world is demanding more expensive necessities of life which the physician cannot dispense with if he is to maintain his position and move with the current of the time.

It is obvious that a man cannot do everything in one short life, and so our investigations and practice are distributed along lines that are becoming more and more special. We are dividing more our work and are uniting more our counsel, and in this fact will be found one of the causes that are turning people towards hospitals and public clinics. It is even now unusual for a diagnostician to satisfy himself on all sides of a complicated case without calling to his assistance the special knowledge of one or more of his colleagues. He may require blood cultures or urine examinations that involve proficiency in organic chemistry, or he may desire the opinion of a surgical colleague. The well-to-do should pay for each and all of these examinations, whatever may be necessary, but such a course is impracticable for people of small means, yet upon going into a hospital they are able to receive an opinion made more valuable because of more complete study, and this without a charge that is prohibitive. I can readily understand how this course is for the best interest of persons of small means, but also it is apparent that it curtails the income of physicians. It is well enough to say that a young man must develop himself for higher work or perfect himself in some special subject, but such a course will eventually fail without opportunity for practice.

A physician cannot avail himself of the possibilities that outreaching science holds in store without means of support. With most of us the available means of support are to be found in our work and there only, and if a large proportion of the work is to be carried on by hospitals, dispensaries, and a sort of impersonal physician employed by insurance companies, workshops and various altruistic organisations, we must ask ourselves seriously how are we to survive in the future, how successfully to keep pace with these competitors of our own creating? It will not do to say that this is a passing social phase, and that it will soon disappear, for if we examine the situation critically we shall discover that these problems are evidences of a social revolution that is only fairly begun, that will inevitably continue with grownig momentum until a great bulk of disability will be treated in institutions, or by experts who occupy a semi-official position, similar to those hitherto mentioned. Silently and swiftly this movement is going on by the unconscious help and furtherance of many physicians who today may deny its very existence; and it appears that the development of which the Boston Medical Society now complains is merely a foretaste of what the future has in store for us.

Does this imply that the private physician, "the doctor," in the sense in which for centuries he has been regarded, is to disappear from the community? In my judgment the answer must be decidedly in the negative. Individualism in medicine is absolutely necessary, and without the private worker it is likely that the very framework of medicine would be weakened. Not only will there be the private physician, but he will be better, more effectual than the physician of today. Possibly physicians will be fewer in number. This may depend in part upon the increased expenditure of time and money in fitting for the work, and perhaps upon the reason that there will not remain a sufficiently wide clientele for the present large proportion of private practitioners. However, this is by no manner of means sure. All of these new movements in the semi-public method of dealing with the problems of disease will require the intelligence and the service of a large number of workers. The field of labor must be greatly widened, for although, from prevention, illness will be made to decrease, this can only be effected through the application of preventive medicine in its widest sense. Such matters as pure water and food supply, ventilation, recreation, exercise, and the like, may be carried on without great demands upon the time of the physician, but it is evident that other fields will open in which work must be conducted by highly trained and discriminating professional men. For it will be admitted that new causes of disease are increasing with the disappearance of old causes like, for instance, infected drinking water. We need not look far to discover modern breeders of disability. Life is becoming steadily more complicated and strenuous. Hurry, lack of rest and systemic strain, pressure in modern education and social exactions are sure to produce their victims. Besides this there is a growing tendency to discover and present for needed treatment a goodly number of unphysiologic conditions that hitherto have largely escaped notice, such as weak feet, crooked spines, adenoids, eye-strain, and mental peculiarities; also expertness in the use of the increasing number of instruments of precision and the rapid extension of laboratory work are certain to open new and wide fields for the employment of the physician.

It therefore seems that the occupation of the physician will not cease because of the disappearance of disease from the planet, and merely in the intelligent prevention of these affections there of necessity will be found a place for the activity of the best minds. When we subject the matter to close examination it appears that we are in the rapids of an extraordinary social change, and that in order to navigate safely we must change our methods, but not our calling. It is to be hoped that we shall be more occupied in the prevention than in the cure of disease, and it is doubtful if we shall have less occupation or be more poorly compensated. The times are changing, and therefore we must change, for experience teaches that the man who is out of harmony with the spirit of his times is inevitably left behind and forgotten. In all the history of medicine the place of the doctor has undergone little variation, but we have not before come upon times like these, and in dealing with the new economic problems which are presenting themselves we must be foresighted and prepared, whereupon we shall have the satisfaction of contributing largely to the welfare of the race, of enjoying the reward of usefulness, and of close relationship with principles of the deepest interest.

The profession of law has undergone a transformation within the last quarter of a century. The old-fashioned lawyer is now rarely seen, for he has been succeeded by the modern lawyer-business man who deals with affairs from a different standpoint than did his predecessor. While this transition was in process many good lawyers of the old school found themselves outdistanced by the more progressive and business-like men who more quickly adapted themselves to the changing relations. It is not unlikely that some of us shall suffer in much the same way.

No one can look upon the present situation without a feeling of sadness. There is an increase in the profession of relatively highly trained men who are finding it impossible to achieve a competency in practice. At the same time many who are well established feel themselves encroached upon, pushed into narrower and narrower straits.

With all great social changes there are borne forward on the wave of popularity new desires, new necessities and new preoccupations, while old, valuable, and even sacred ideals disappear as in the hollow of the sea. So it was in the Augustan age at Rome, in the decay of the ancient regime in France, and as we now see it in the extraordinary evolution in China, where at present there is unfolding a more miraculous and sudden transformation of the ideals and customs of a people than is elsewhere recorded in history. We may imagine a day when some Chinaman, wearing the habiliments common to Hongkong and Manchester, standing upon the holy mountain of Confucius, shall gaze with sad, regretful eyes across that vast expanse, the ancient realm of China, while the ghosts of countless ancestors weigh down his understanding and point backward to a thousand endeared and revered institutions disappearing in oblivion. It is natural to regret old and dying customs; to wipe out an honored tradition is to injure confidence in realities, unless the old is replaced by a doctrine that is manifestly uplifting to civilisation. In the further working out of this problem of ours we are destined to suffer the loss of some of our professional characteristics, but we shall achieve new ones. We are likely to recall with regret some of the abandoned prerogatives of the earlier days, but indifferent to our sentiments, the further working out of the genius of modern civilisation is inevitable, quite regardless of the benefits or hardships that will ensue. "Such is the eternal law of life, ceaselessly transforming good into evil and evil into good." (G. Ferrero).

In view of what has been said, what should be the attitude of our profession toward this question? We can do one of three things: First, help it on, as many are doing; second, oppose it, which is being done by some; third, we may look on. Of the first, that of lending assistance, it appears to be the natural and progressive course. We shall be drawn more and more in its wake until it comes to be the accepted creed of medicine. There never has been a time when our profession has not stood for the good of the race. When individual members have lost sight of this fact they have sloughed off from the proper body of the profession. The prospective change in relations will be difficult for some men who may not have the natural aptitude for the new undertakings, and for those who are too old or too weak to undergo the strain of the transition. It is to be hoped that those who elect to be onlookers will observe with the spirit of philosophers and leave for posterity an illuminated record of this remoulding of the profession.

Those who choose to oppose the movement may be warmed by a sentiment of loyalty to time-honored conditions and may never realise their defeat. It is often so with certain temperaments, as has been exampled so often in religion and politics. It is said of certain men that they still believe that Thomas Jefferson is living.

It would seem to be a wiser plan deliberately to coöperate with the movement, to curb its rank and unintelligent exuberance where we may, to cement in a lasting foundation that which is undeniably good. To this end it is necessary now, more than ever before, for the physician to interest himself in sociology as well as pathology, and thus to create for himself a larger place, more amplitude of vision, a wider interest, and the means of subsistence. There must necessarily be a demand for our special training, and it will probably be found that we shall have no less active occupation when we shall have adapted ourselves to these new requirements of a contending civilisation.

It is highly probable that the aroused public interest in the subject of medicine will result in a much more enlightened laity than we have previously known. Many diseases may be prevented, or their on-coming foreseen, so that the physician will have more opportunity for treating the patient rather than the disease. The great question of heredity may eventually receive discriminating attention. But long before this it is probable that methods will be found more economical in suffering and expense than is at present true in the management of a large proportion of crippled and neurasthenic, of inebriates and others who now encumber society. But even though this tendency of the semi-public treatment of disease appears to be temporarily an obstruction to the profession, even though in some respects it is found to be inimical to our interest and destructive to the welfare of the laity, it is sure in the course of events to right itself, leaving for us a residuum of good, as has always been true in the annals of medicine.

This movement is now on trial. That portion of it that proves to be for the best interest of all will survive and expand as have other innovations in the past. In conforming with new ideals we shall not be doing the unprecedented in our history. For instance, certain schools that in the past have agitated the placid surface of self-satisfaction really forced themselves upon our attention. These we resisted until whatever truths they concealed were disclosed, whereupon we embraced the kernels of truth and we have survived to see the disappearance of so-called heresies, for an idea ceases to be heretical when its truth is discovered.

In this way we have come to know the facts of small medication, of psychotherapy, of balneology, and of medical gymnastics. We have been not harmed but helped by these vicissitudes, not weakened but strengthened. It is the happy lot of medicine that it stands for truth wherever it may lead, and hence it is safe. Time and experience see the expiring error leaving a small fruitage of reality, all that was borne from the exuberance of stalk. Therefore we need not worry about the future in medicine. Whatever in this new tendency may prove to be wise and economical we shall adopt and practice. Ultimately much of it may be left in oblivion, but not until it has been tested by a far wider application than we have yet seen. Meantime those who insist upon the survival of old privileges will meet with what appears to be an ungrateful opposition, and, what is saddening to reflect upon, many worthy men will find the later years of life unduly cramped.

Always the fear of poverty and dissolution results in timid action and inhibits success. Therefore let us meet this new problem with the spirit of true philosophy. I admire this passage from Montaigne: "A true conquest respecteth rather an undaunted resolution, an honorable end, than a fair escape; and the honor of virtue doth more consist in *combating* than in *beating.*"

Therefore, instead of emphasising our privileges and asserting too loudly our rights, let us rearrange our molecules and push on. Then we are likely to rise to a higher plane of usefulness and greater satisfaction. This will not come by attempting to obscure the dawn of better things, but by helping to dispel the mists, making more clear the light in which civilisation must journey towards truth.

